# A voxel-based morphometry study of disease severity correlates in relapsing–remitting multiple sclerosis

**DOI:** 10.1177/1352458509351896

**Published:** 2010-03

**Authors:** A Prinster, M Quarantelli, R Lanzillo, G Orefice, G Vacca, B Carotenuto, B Alfano, A Brunetti, V Brescia Morra, M Salvatore

**Affiliations:** 1 Biostructure and Bioimaging Institute, National Research Council, Naples, Italy.; 2 SDN Foundation, Naples, Italy.; 3 Department of Neurological Sciences, University ‘Federico II’, Naples, Italy.; 4 Neurology, Hermitage Hospital, Capodimonte, Naples, Italy.; 5 Department of Biomorphological and Functional Sciences, University ‘Federico II’, Naples, Italy.; 6 Neuroradiology, University ‘Federico II’, Naples, Italy.

**Keywords:** multiple sclerosis, relapsing–remitting, voxel-based morphometry, Expanded Disability Status Scale, lesion load, cortical atrophy

## Abstract

Previous studies have shown a preferential loss of grey matter in fronto-temporal regions in patients with multiple sclerosis. Studies of correlates of disease severity are more controversial, because some studies have suggested an association between sensorimotor cortex atrophy and Expanded Disability Status Scale score, while others did not find such a correlation. The objective of this study was to assess the correlation of regional loss of grey matter and white matter with indexes of clinical and radiological severity in relapsing–remitting multiple sclerosis, including the Expanded Disability Status Scale and lesion load. Correlations between Expanded Disability Status Scale, lesion load and disease duration were assessed in 128 patients with relapsing–remitting multiple sclerosis (Expanded Disability Status Scale range 1.0–6.0) using optimized voxel-based morphometry. Bilateral loss of grey matter in sensorimotor cortices was correlated with Expanded Disability Status Scale, and tissue loss also involved adjacent white matter, extending along pyramidal tracts to the brainstem. Increasing lesion load was correlated with loss of deep grey matter and white matter. No specific region of grey matter or white matter showed a significant correlation with disease duration. These findings support the hypothesis that motor neuron involvement plays a major role in the progression of physical disability. Lesion load accrual affects mainly highly interconnected subcortical structures, while disease duration has a less significant impact on brain atrophy, probably owing to the inter-subject heterogeneity of the clinical course of the disease.

## Introduction

The results from previous studies of regional brain tissue loss in multiple sclerosis (MS) suggest that grey matter (GM) atrophy, in patients affected by relapsing–remitting (RR-)MS, is located preferentially in specific regions of the brain, including motor areas^1,2^ and thalami.^3^ Few studies have addressed the correlations between regional brain atrophy and indexes of disease severity, including disability and lesion load (LL), and they provide somewhat conflicting results.^1,2,4–6^

In particular Chen et al.^5^ and Sailer et al.,^2^ in two small groups including both RR and secondary progressive (SP-)MS patients, found a significant thinning of the motor areas in patients with more severe disability. However, other studies focusing on RR-MS were not able to detect significant correlations between motor cortex atrophy and clinical disability in small groups of patients,^1,6^ or in the early phases of the disease.^4^

When assessing the relationship of atrophy with LL, Sailer et al. found a significant thinning of the prefrontal cortex in a subgroup of patients with higher LL, as compared to the control group.^2^ Charil et al. demonstrated a preferential correlation of cortical thickness with T2-LL in bilateral anterior cingulate gyrus, bilateral posterior insular cortex in the vicinity of the transverse temporal gyrus, right anterior and middle temporal gyri and left inferior frontal and orbital gyri.^4^ Other groups have shown a significant correlation between LL and GM volume in the caudates,^1^ the frontal and parietal cortices in a longitudinal study,^7^ and thalami in transversal studies.^6^

All these attempts at correlating regional brain tissue loss and disease severity have focused on GM volume or cortical thinning, while correlates of regional white matter (WM) volume have not been assessed, although WM loss has been demonstrated in different cohorts of MS patients,^8,9^ and may play a role in determining disease severity.

Voxel-based morphometry (VBM),^10^ a fully automated technique used to assess the density of brain tissues at a voxel level, has been applied previously to assess the regional distribution of GM loss in RR-MS,^1,3,6,7^ and is suitable for the study of changes in the volume of both GM and WM.

The aim of the present study was to assess the correlation between regional losses of GM and WM and indexes of disease severity, including clinical disability (as measured by the Expanded Disability Status Scale, EDSS) and LL, in a large group of patients with pure RR-MS, using VBM.

## Materials and methods

### Participants

One-hundred and twenty-eight patients (86 female) with MS, clinically defined according to Poser criteria,^11^ and with an RR course^12^ were recruited into the study. The mean age of the patient group was 36.07 ± 9.2 years (range 18–58 years), with a mean disease duration (DD) of 10.14 ± 7.2 years (range 0.3–30.8 years) and a median EDSS score of 2.8 (range 1.0–6.0).^13^

The exclusion criteria for this study included treatment with corticosteroids within the preceding 28 days, previous treatment with cyclophosphamide, azathioprine, methotrexate, mithoxantrone or cyclosporine, or previous treatment with any experimental immunomodulating drug.

At the time of the scan 13 patients were drug naive, while the remaining patients had for at least six months been given interferon beta (108 patients on interferon beta-1a, 7 on interferon beta-1b).

Thirty-five healthy participants (18 female) were also included in the analysis for comparison. The mean age of the normal volunteer (NV) group was 38.31 ± 10.97 years (range 22–57 years). All subjects agreed to participate in the study by signing a written informed consent, and the ethical committees of the participating institutions had previously approved the protocol.

### Magnetic resonance imaging studies

The magnetic resonance imaging (MRI) protocol included two interleaved sets of 16 slices each covering the whole brain, which were obtained at 1.5 T (Intera, Philips Medical Systems, the Netherlands), sampling the brain at a total of 32 contiguous levels. Each of the two sets included conventional spin-echo sequences providing T1w (520/15 ms TR/TE) and PD/T2w (1800/15–90 ms TR/TE) 4 mm thick axial images (24 cm FOV, 256 × 256 acquisition matrix).

### Voxel-based morphometry analysis

The MRI volumes were preliminarily segmented into GM, normal and abnormal WM, and cerebrospinal fluid (CSF) using a fully automated relaxometric method described in detail elsewhere,^1,14^ which provides the binary maps of normal-appearing GM, normal-appearing WM, abnormal WM (providing the T2-w lesion load) and CSF. A previous work that assessed the accuracy of segmentation of both normal and abnormal tissues demonstrated a lack of a LL-associated bias in the measure of brain tissue volumes (100% recovery of GM volume, with a non-significant correlation between GM volume and LL).^1^

The volumes of GM and total WM (hereinafter WM indicates the sum of both normal-appearing and abnormal WM) were analysed, using optimized VBM analysis,^10,15^ to assess both regional differences between the NV and MS groups, and correlations with EDSS and LL in the MS group.

The VBM was performed using the Statistical Parametric Mapping software (SPM5, Wellcome Department of Cognitive Neurology, London, UK, http://www.fil.ion.ucl.ac.uk/spm)^16^ running under MATLAB version 6 (the Mathworks, Inc., Massachusetts, USA). Details of the protocol for optimized VBM as applied to segmented volumes has been described in detail elsewhere.^1^

Briefly, for each of the two analysed types of brain tissue (GM and WM), preprocessing included the following steps:

–Normalization of the GM and WM maps of each participant to the MNI (Montreal Neurological Institute) space using the SPM5 mean GM and WM templates with a 12-parameter affine model without any nonlinear component.^17^ Nonlinear components were not used for template creation in order to preserve group affine geometry.^18^
–Creation of GM and WM templates specific for site and group, which were obtained by averaging the corresponding roughly normalized brain tissue volumes obtained in the previous step, and by subsequent smoothing with a Gaussian filter of 8 mm full-width at half-maximum (FWHM).
–Non-linear normalization of the original GM and WM maps to the corresponding site- and study-specific templates with 16 non-linear iterations using 6 × 8 × 6 basis functions to account for global shape differences.^19^ Normalized images were resampled by trilinear interpolation to 1 × 1 × 1 mm^3^ voxel size.
–The resulting normalized images were then modulated by multiplying the GM and WM voxels by the Jacobians derived from the corresponding spatial normalization parameters, to preserve the amount of the specific tissue in each voxel.^10^
–Finally, the modulated images were smoothed with a 12 mm FWHM isotropic Gaussian filter to reduce confounding by individual variations in gyral anatomy and to render the data more normally distributed as per the Gaussian random field model underlying the statistical process used for adjusting *p*-values.^17^

### Statistical analysis

The effect of the disease on global brain tissue volumes was preliminarily tested, separately for GM, WM and CSF, by stepwise multiple regression analysis using SPSS (SPSS Inc., Chicago, Illinois, USA). Total intracranial volume (ICV, the sum of GM, WM and CSF), age and sex were entered first into the model to account for the possible effects of these variables. Subject group (NV versus MS) was then entered to explore the effect of the disease. Subsequently, LL, EDSS and DD were entered to test for the possible effect of these variables on global brain tissue volumes in MS patients. The significance level was set to 0.05, and corrected for multiple comparisons according to Bonferroni.

To search for regional correlations between brain tissue volumes and clinical variables, normalized modulated GM and WM volumes were analysed separately at voxel level using a generalized linear model, with correction for multiple comparisons based on the random Gaussian field theory,^16^ including age and sex as nuisance regressors (confounding covariates) in an analysis of covariance (ANCOVA), to take into account their effect on brain tissue volumes. The ICV was included in the model to normalize for head size.

Prior to regression analysis, scans were thresholded at a fixed intensity value of 25 to reduce the influence of any remaining non-brain tissue. Correlations for GM and WM were assessed separately versus group (NV or MS), EDSS, LL and DD. For each correlation both direct and inverse contrasts were probed. A *p*-value of 0.05 FWE Family-wise error-corrected at voxel level was used for comparisons between NV and MS patients.

Correlations between GM or WM and EDSS, LL and DD were examined only in regions found previously to be abnormal in the MS patient group. For this purpose, an inclusive mask was built using the control versus patient statistical map with a more lenient statistical threshold of *p* < 0.05, corrected for false discovery rate.^20^ Within this mask, the clusters were considered significant at *p* < 0.05, corrected for multiple comparisons at the cluster level.

## Results

The mean LL was 6.8 ± 9.28 cc (mean ± SD, range 0–46.0 cc). Analysis of the global tissue volumes of our patient population showed significantly decreased GM volume (45.9 ± 3.7% and 47.6 ± 3.7% of ICV respectively for MS patients and NV, *p* < 0.05 on multiple regression analysis) and WM volume (37.1 ± 3.1% and 38.9 ± 2.2%, *p* < 0.005), as compared with NV, and a correspondingly significant increase in CSF (17.0 ± 5.4% and 13.5 ± 3.3%, *p* < 0.0005).

When assessing the correlation of global tissue volumes with clinical parameters, we found a significant linear correlation of GM loss with LL (*p* = 0.001), while it only approached significance when tested versus DD (*p* = 0.04) and EDSS (*p* = 0.06). The WM loss correlated significantly with DD (*p* < 0.01), LL (*p* < 0.00005) and EDSS (*p* < 0.0001). The volume of CSF correlated with DD (*p* < 0.005), LL (*p* < 0.000005) and EDSS (*p* < 0.0005).

Table 1 reports the clusters of significant GM loss in MS patients, compared with NV. For each cluster, the extension and the corresponding Brodmann Areas (BA) in which local maxima are located, along with the coordinates in the Talairach space,^21^ and the T level of the most significant voxel are reported.

**Table 1.** Clusters of significant grey matter loss in patients with relapsing–remitting multiple sclerosis relative to controls. For each cluster, the extension and the corresponding Brodmann areas (BA) in which local maxima are located, along with the coordinates in the Talairach space and the T level of the most significant voxel, are reported

					Coordinates (mm)		
Region	Side	BA	*N. voxel*	*T*	*x*	*y*	*Z*
Precentral gyrus	L	4/6	52739	7.50	−30 −32 −35	−26 −20 −13	55 12 45
Insula/claustrum	R	13	3045	7.07	33 44	−18 −9	14 0
Caudate head	L/R	n/a	1267	6.85	−3 −7	10 17	3 2
Cingulate gyrus	bilateral	24/31	10571	6.28	−6 5 −9	24 31 −23	21 13 37
Middle frontal gyrus	R	8	1298	6.13	23	18	39
Middle frontal/precentral gyrus	R	9/8	4743	5.95	45 39 41	12 26 24	33 48 38
Precentral/postcentral gyrus	R	6/3	1285	5.47	37 42	−6 −16	38 51
Inferior/superior Parietal lobule	R	40/7	2027	5.36	40 36 34	−41 −48 −47	53 47 61
Superior temporal gyrus	L	9/8	367	5.05	−48	−52	30

n/a, not applicable.

**Figure 1.**
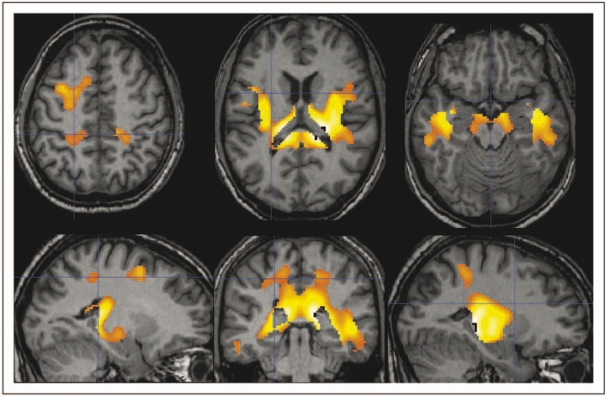
Axial sections (first row, patient’s left side is on the observer’s left side), sagittal sections of the left (bottom left) and right (bottom right) hemispheres, and coronal section (bottom row, centre) from selected levels of a normal subject T1-weighted volume (in greyscale). The regions of significant white matter loss in patients with multiple sclerosis as compared with controls are overlaid in colourscale. Bilaterally, in the periventricular regions in the temporal lobes, juxtacortical insular regions, extending posteriorly through the internal capsule to the thalami, and the splenium of the corpus callosum are involved.

In RR-MS, loss of GM involves preferentially the left fronto-temporal cortex and precuneus, as well as the anterior cingulate gyrus and the caudate nuclei bilaterally, and to a minor extent the right fronto-temporal cortex and right parietal lobule. The VBM analysis of WM indicated preferential areas of WM loss in RR-MS bilaterally in the periventricular regions in the temporal lobes, juxtacortical insular regions, extending posteriorly through the internal capsule to the thalami, and to the splenium of the corpus callosum (Figure 1). The inverse contrast (MS greater than NV) did not result in any significant region for either GM or WM.

**Figure 2.**
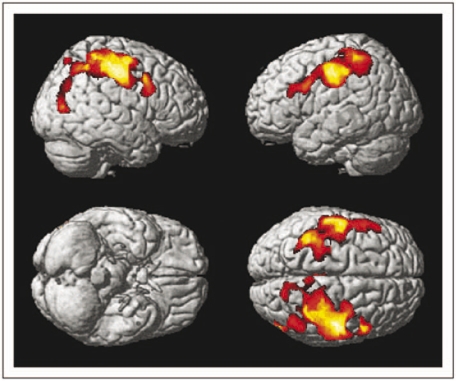
Regions exhibiting a significant correlation between grey matter loss and Expanded Disability Status Scale (*p* < 0.05 corrected for multiple comparisons at cluster level, i.e. *t* score >3.16) in patients with relapsing–remitting multiple sclerosis are overlaid in colour on to the surface of a single subject normalized brain.  The primary motor and somatosensory areas, and the middle frontal gyri are involved bilaterally, with extension to the middle temporal gyrus in the right hemisphere.

Regions of GM loss that showed a significant linear correlation with EDSS are shown in Figure 2 and summarized in Table 2, and include the primary motor and somatosensory areas and the middle frontal gyri bilaterally, with extension to the middle temporal gyrus in the right hemisphere.

**Table 2.** Clusters of significant grey matter loss that correlate with Expanded Disability Status Scale in patients with relapsing–remitting multiple sclerosis. For each cluster, the extension and the corresponding Brodmann areas (BA) in which local maxima are located, along with the coordinates in the Talairach space and the T level of the most significant voxel, are reported

				Coordinates (mm)		
Region	Side	*N. voxel*	*T*	*x*	*y*	*Z*
Precentral gyrus/postcentral gyrus	R	41162	4.83	37 26	−32 −24	53 52
Frontal lobe – paracentral lobule	R			13	−37	44
Precentral gyrus/postcentral gyrus	L	18416	4.73	−53 −44	−15 −15	34 40
Inferior parietal lobule	L			−40	−39	57

**Figure 3.**
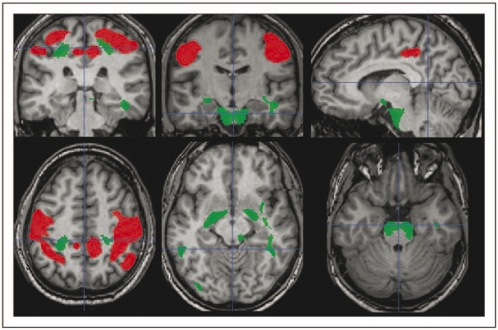
Regions of white matter loss (in green) that correlate with Expanded Disability Status Scale in patients with relapsing–remitting multiple sclerosis together with regions of grey matter loss (in red) for comparison (*p* < 0.05 corrected for multiple comparisons at cluster level, i.e. *t* score >3.16). White matter loss is located bilaterally in the juxtacortical regions adjacent to the primary motor cortex and lower temporo-parietal regions, in the brainstem (both pyramidal tracts and tegmentum), and in the anterior part of the corpus callosum.

In Figure 3 is shown the map of the regions, indicating a significant correlation between WM loss and EDSS (in green) along with the regions of GM loss correlating with EDSS (in red). Regions of WM loss correlating with EDSS are located bilaterally in the juxtacortical regions adjacent to the primary motor cortex and lower temporo-parietal regions, in the brainstem (both pyramidal tracts and tegmentum) and in the anterior part of the corpus callosum.

**Figure 4.**
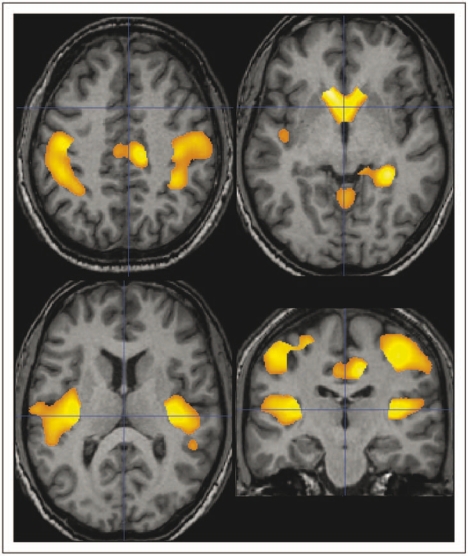
Regions of significant correlation between grey matter loss and lesion load (*p* < 0.05 corrected for multiple comparisons at cluster level, i.e. *t* score >3.16) in patients with relapsing–remitting multiple sclerosis are overlaid in colour on to the sections of a normal subject T1-w images and are symmetrically distributed in the caudate heads, the parahippocampal and cingulate gyri, motor cortex and insula.

Correlations between regional GM loss and lesion load (LL) are depicted in Figure 4 and summarized in Table 3. Regions of GM loss that correlate significantly with LL are distributed symmetrically in the caudate heads, the parahippocampal and cingulate gyri, motor cortex and insula.

**Table 3.** Grey matter clusters exhibiting a significant negative correlation with lesion load in patients with relapsing–remitting multiple sclerosis. For each cluster, the extension and the corresponding Brodmann areas (BA) in which local maxima are located, along with the coordinates in the Talairach space and the T level of the most significant voxel, are reported

					Coordinates (mm)		
Region	Side	BA	*N. voxel*	T	*x*	*y*	*Z*
Caudate head	L/R	n/a	8284	7.20	−10 6	19 15	2 2
Parahippocampal gyrus	L/R	30/36	8375	5.95	18 −10	−43 −38	−13 −10
Cingulate gyrus	L/R	31/24	5444	5.86	11 −4	−21 −16	36 36
Precentral/postcentral gyrus	R	3/4	19487	5.59	39 48	−21 −13	48 48
Insula Transverse temporal gyrus	L	13 42	8733	5.55	−35 −64	−15 −16	15 10
Insula Postcentral gyrus	R	13 40	5574	5.50	36 52	−20 −28	11 20
Precentral gyrus	L	4/6	16980	5.0	−26 −44	−11 −16	51 41

**Figure 5.**
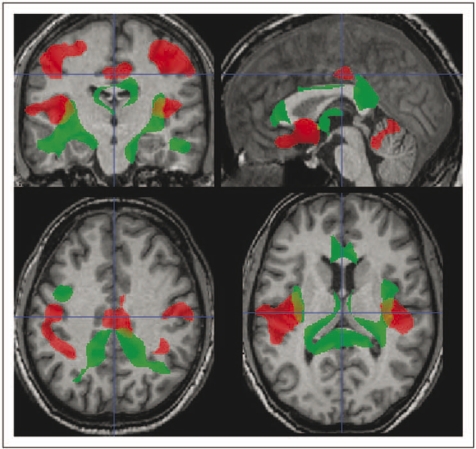
Regions of white matter loss (in green) that correlate with lesion load in patients with relapsing–remitting multiple sclerosis together with regions of grey matter loss (in red) for comparison (*p* < 0.05 corrected for multiple comparisons at cluster level, i.e. *t* score >3.16). They include the corpus callosum, with more extensive involvement of the genu and splenium, the latter extending bilaterally to the juxtacortical fibres abutting the precuneus/posterior cingulate, thalamocapsular white matter, and the deep temporal white matter bilaterally.

Regions of WM loss that correlate with LL are reported in Figure 5. These include the corpus callosum, with more extensive involvement of the genu and splenium, the latter extending bilaterally to the juxtacortical fibres abutting the precuneus/posterior cingulate, thalamocapsular WM, and the deep temporal white matter bilaterally.

Assessment of the correlations between either GM or WM loss and DD did not result in any significant cluster.

## Discussion

The aim of this work was to assess the possible correlations between regional GM and WM loss and clinical parameters, including EDSS score (an index of disability), total LL (an index of disease severity) and DD, in a large group of patients with RR-MS.

The demonstration of global reduction in GM and WM volumes in RR-MS is in line with previous findings of GM and WM atrophy,^22^ and the preliminary assessment of regional GM volume in RR-MS, as compared with NV, confirmed previous findings, obtained using the same optimized VBM approach in a smaller patient group, of a preferential involvement of frontal regions.^1^

Additional assessment of WM volumes showed a more widespread loss, involving, besides periventricular and medial temporal WM (reflecting ventricular enlargement, which is predominant in RR-MS),^23^ pyramidal tracts extending from juxtacortical fibres adjacent to the sensorimotor cortex, through thalamo-capsular regions to the cerebral peduncles.

The significant loss of brain tissue in the thalamic region, with a significant correlation with LL, may actually reflect brain tissue loss in both pyramidal and thalamic regions, because with the present segmentation technique most of the lateral thalamus is segmented as WM. Thalamic atrophy has been found in many studies of MS, and seems to be present already in the very early stages of MS, including clinical isolated syndrome (CIS).^24^

Give that the thalami are composed of GM nuclei interspersed among WM tracts, classification even with a multiparametric approach is challenging, and studies focusing specifically on this region, e.g. using diffusion tensor-based segmentation,^25^ are required to disentangle the relative contributions of these anatomical structures to local loss of brain tissue.

Previous work probing the correlations between global brain tissue volumes and indexes of disease severity have shown that global GM loss correlates weakly with EDSS in RR-MS in transversal studies,^26^ while a few longitudinal studies have suggested that patients who show sustained progression in disability (as measured by EDSS) present a significantly faster progression of the atrophy compared with patients with relatively stable disease.^27–29^ In our patient population we confirmed the presence of a correlation between EDSS and global brain atrophy, as measured by increased CSF, which was sustained by a reduction of global WM and (to a lesser extent) GM volumes.

When testing correlations of regional loss of GM with disease severity using VBM, motor areas showed a preferential correlation with EDSS bilaterally. These results confirm and extend previous observations from studies of cortical thickness, in small groups of MS patients with mixed disease course, that suggested a reduction in regional cortical thickness at the level of the motor areas in patients with higher disability scores as compared with NV,^2^ with a rate of GM thickness reduction that was more pronounced in the parietal and precentral cortex in patients with a more aggressive disease course, compared with stable patients.^5^

A previous study in a smaller cohort of patients with pure RR-MS, which used the same methodology as the present study to analyse regional GM volume loss, failed to detect significant regional correlates of EDSS.^1^ This was probably attributable to the reduced sample size and the smaller span of EDSS values (51 patients with an EDSS range of 1.5–4.5, versus 128 patients with EDSS range 1.0–6.0).

Another study that focused on correlates of regional GM loss in a large cohort of patients with pure RR-MS, using a technique based on the assessment of cortical thickness to look for correlations with LL and disability in 425 patients, found a higher rate of thinning per unit increase in EDSS in the prefrontal regions bilaterally and in the anterior cingulate regions.^4^ The authors suggested that the poor correlation of cortical thinning of the premotor and motor areas with EDSS and LL might be due to the relatively mild impairment of their patients, with a high prevalence of patients at relatively early stages of the disease in the sample.

The present data, however, do not support this hypothesis because correlation of primary sensorimotor areas with EDSS was present and sizeable, despite the smaller EDSS range (1–6 as compared to 0–8 in the cohort published by Charil^4^) and smaller LL (up to 46.0 cc, compared with 78.6 cc).

Differences in the techniques used to assess atrophy (cortical thickness versus VBM) might contribute to the different results. In particular, studies of cortical thickness may be more sensitive to variations in GM regions with stronger morphological variability, such as the frontal cortex,^30,31^ while more stable regions of the brain may be better explored using VBM. However, to the best of our knowledge, comparative studies using both techniques are not currently available, and further work is needed to assess differences between these two techniques.

Additionally, criteria for patient selection may have played a role in determining different results; for example, current use of disease modifying therapy (DMT) was not an exclusion criterion in the present study, while previous use of immunosuppressors or experimental immunomodulatory therapies was not an exclusion criterion in the aforementioned paper.

Regarding correlation of GM loss and LL, the current results extend the previous findings of a correlation between GM loss and LL at the level of both caudate heads, showing areas of significant correlation also in the cingulate gyri, sensorimotor regions and temporal lobes, including the insulae.^1^

Interestingly, deep GM atrophy has been related to global LL also in primary and secondary progressive forms of the disease, as opposed to regional cortical atrophy which has been related in the same forms to corresponding lobar lesion loads.^6^

In that same study, however, Ceccarelli et al.^6^ demonstrated in RR-MS a correlation of thalamic atrophy with LL, which, owing to the differences in the segmentation techniques, may be reflected partly by the significant clusters that were detected in the present study in the thalamo-capsular regions in the VBM analysis of the WM. Also, differences in data acquisition may contribute to different sensitivities. For the present work we used segmented two-dimensional sequences, which in principle may limit the accuracy of the method as compared to the use of three-dimensional sequences with isotropic resolution. However, it should be considered that the preprocessing for VBM involves a smoothing three times larger than the slice thickness (12 mm FWHM), thus making the use of 4 mm sections unlikely to affect the results of VBM.

The use of VBM to assess regional loss of WM allowed demonstration of a significant correlation with EDSS in the WM, which stemmed from the motor areas bilaterally and followed the pyramidal tracts down to the brainstem, thus supporting the hypothesis that motor neurons are more severely damaged as disability progresses.

In RR-MS, a correlation between motor disability and spinal cord atrophy has been previously demonstrated,^32^ which may be in turn related also to atrophy of the pyramidal tracts. However, further work is needed to explore this possible mechanism, and to assess possible specific clinical correlates of pyramidal tract atrophy.

It is of note that DD showed a weaker correlation with global atrophy, compared with LL and EDSS, without any regional specificity in the VBM analysis. This may be due to both the difficulty in defining the time of disease onset based on the anamnesis, as well as the intrinsic inter- and intra-subject variability of disease progression in RR-MS.

A limit of our study lies in the heterogeneity of the patient group in terms of clinical variables, which facilitated the detection of potential correlates of EDSS and DD, but in turn limited the current approach to the study of the common patterns of brain tissue involvement across a heterogeneous population, while correlations that may be present only in MS subgroups (e.g. early MS, benign MS) may have remained undetected.

Furthermore, the presence of atrophy in the early phases of the disease, including CIS,^24^ may limit the sensitivity of correlation analysis. The correction for age, here included both as a first step in the multiple regression analysis of global tissue volumes, and as a nuisance covariate in the VBM analysis, to allow for the strong correlation of brain tissue volumes with age,^15^ may have hindered detection of an effect of DD on atrophy within the constraints of the general linear model generalized linear model.

### Conclusions

In RR-MS, we have confirmed a direct correlation between disease severity and brain tissue loss (both GM and WM) in the motor system, while LL correlates of brain tissue loss mainly affect highly interconnected subcortical structures, including the caudate nuclei and thalami.
